# Quantification of Cellular NEMO Content and Its Impact on NF-κB Activation by Genotoxic Stress

**DOI:** 10.1371/journal.pone.0116374

**Published:** 2015-03-05

**Authors:** Byounghoon Hwang, Funita P. Phan, Kevin McCool, Eun Young Choi, Jinsam You, Adam Johnson, Anjon Audhya, Shigeki Miyamoto

**Affiliations:** 1 Department of Oncology, University of Wisconsin, Madison, Wisconsin, United States of America; 2 Integrated Program in Biochemistry, University of Wisconsin, Madison, Wisconsin, United States of America; 3 Molecular and Cellular Pharmacology, University of Wisconsin, Madison, Wisconsin, United States of America; 4 Department of Biomolecular Chemistry, University of Wisconsin, Madison, Wisconsin, United States of America; 5 Department of Biochemistry and Molecular Biology, Indiana University School of Medicine, Indianapolis, Indiana, United States of America; St. Georges University of London, UNITED KINGDOM

## Abstract

NF-κB essential modulator, NEMO, plays a key role in canonical NF-κB signaling induced by a variety of stimuli, including cytokines and genotoxic agents. To dissect the different biochemical and functional roles of NEMO in NF-κB signaling, various mutant forms of NEMO have been previously analyzed. However, transient or stable overexpression of wild-type NEMO can significantly inhibit NF-κB activation, thereby confounding the analysis of NEMO mutant phenotypes. What levels of NEMO overexpression lead to such an artifact and what levels are tolerated with no significant impact on NEMO function in NF-κB activation are currently unknown. Here we purified full-length recombinant human NEMO protein and used it as a standard to quantify the average number of NEMO molecules per cell in a 1.3E2 NEMO-deficient murine pre-B cell clone stably reconstituted with full-length human NEMO (C5). We determined that the C5 cell clone has an average of 4 x 10^5^ molecules of NEMO per cell. Stable reconstitution of 1.3E2 cells with different numbers of NEMO molecules per cell has demonstrated that a 10-fold range of NEMO expression (0.6–6x10^5^ molecules per cell) yields statistically equivalent NF-κB activation in response to the DNA damaging agent etoposide. Using the C5 cell line, we also quantified the number of NEMO molecules per cell in several commonly employed human cell lines. These results establish baseline numbers of endogenous NEMO per cell and highlight surprisingly normal functionality of NEMO in the DNA damage pathway over a wide range of expression levels that can provide a guideline for future NEMO reconstitution studies.

## Introduction

The Nuclear Factor kappa B (NF-κB) family of dimeric transcription factors regulates gene expression involved in multiple biological processes including immune and inflammatory responses and control of cell proliferation and death [1]. In the absence of stimuli, a NF-κB dimer is kept inactive in the cytoplasm of most cells via association with a member of the inhibitor of NF-κB (IκB) family, which includes IκBα. An important regulator of canonical NF-κB signaling is the IκB kinase (IKK) complex which consists of two catalytic subunits, IKKα and IKKβ, and a regulatory subunit, NEMO (NF-κB essential modulator, IKKγ) [2,3]. Once activated by incoming signals, the IKK complex phosphorylates IκB to stimulate polyubiquitination and proteasome-mediated degradation to release NF-κB [4]. The liberated NF-κB translocates to the nucleus and regulates its target gene expression.

Because the IKK complex represents the convergence point in activating canonical NF-κB signaling, a considerable amount of research has been conducted to understand the mechanism of activation and regulation of the IKK complex. In particular, the role of the non-catalytic subunit NEMO in IKK complex regulation has been studied intensively (reviewed in [2]). These studies have highlighted the role of NEMO as a ubiquitin binding protein to promote IKK activation [5–7]. The recruitment of NEMO to polyubiquitin scaffolds assembled by the upstream signaling events permits the recruitment of the catalytic IKK subunits to the upstream kinase, TAK1 (TGFβ activated kinase 1), which is also recruited to the ubiquitin scaffolds via its ubiquitin binding subunits, TAB2/3, to be phosphorylated and activated [8–10].

In addition to its well accepted role as an IKK regulatory subunit, NEMO also performs an additional upstream role to permit communication between the nuclear DNA damage activated kinase ATM (ataxia telangiectasia mutated) and the cytoplasmic IKK complex to induce NF-κB signaling in response to genotoxic agents (reviewed in [11]). To investigate the distinct functions of NEMO, a variety of mutant forms of NEMO have been previously analyzed [12–15]. However, since overexpression of NEMO can result in inhibition of NF-κB signaling [16–18], it is important to control the amount of NEMO expressed in different cell systems in order to define the various functions of NEMO without being confounded by artifacts associated with high NEMO expression levels. Even so, it is generally undefined what the physiological levels of NEMO are, i.e., how many NEMO molecules are expressed in different cell systems, and what the impact of different expression levels of NEMO is on NF-κB activation by different stimuli, including genotoxic agents.

To answer these questions, purified recombinant full-length NEMO is needed to provide standards for quantification of cellular NEMO levels. Since purification of soluble, recombinant full-length NEMO from *E*. *coli* in high concentrations is technically challenging [19], we first optimized a NEMO purification protocol from *E*. *coli*. We then quantified the average number of NEMO molecules per cell using 1.3E2 NEMO-deficient murine pre-B cell clone stably reconstituted with full-length human NEMO (C5) previously characterized by our lab [12,13]. Next, we generated 1.3E2 clones stably expressing different amounts of NEMO to determine the impact of known numbers of NEMO per cell on NF-κB activation by the DNA damaging agent etoposide, which creates DNA double strand breaks via inhibition of topoisomerase II. Our study demonstrates that a 10-fold range of NEMO levels has surprisingly little impact on NF-κB activation by etoposide and suggests that mutant NEMO phenotypes can be analyzed accurately as long as the level of NEMO remains within this range. We also used the C5 cell line to determine the average number of NEMO molecules per cell in several human cell lines often employed in NF-κB signaling studies to highlight the utility of the C5 cell line as a standard to determine the cellular content of NEMO proteins in different cell systems.

## Materials and Methods

### Reagents

The following antibodies were used: antibody against NEMO (FL-419, sc-8330, Santa Cruz Biotechnology), Flag-HRP (A8592, Sigma), Tubulin (CP06, EMD Millipore), Actin (sc-1616, Santa Cruz Biotechnology), and IKKα/β (sc-7607, Santa Cruz Biotechnology). Anti-rabbit and anti-mouse antibodies conjugated to horseradish peroxidase were obtained from GE Healthcare. Etoposide (E1383, Sigma) and bacterial LPS (L2880, Sigma) were purchased from Sigma-Aldrich. Glutathione-agarose beads and Halt Protease Inhibitor Cocktail was obtained from Thermo Scientific.

### Purification of recombinant full-length human NEMO protein

To purify recombinant full-length human NEMO, NEMO/pGEX6p-1 plasmid was transformed into Rosetta2 BL21 cells and a transformant was grown in 20 ml of LB medium supplemented with 100 μg/ml ampicillin and 34 μg/ml chloramphenicol at 37°C for overnight. The overnight seed culture was diluted in 2 L of LB medium containing 100 μg/ml ampicillin and 34 μg/ml chloramphenicol and grown at 37°C until the absorbance at 600 nm reached 0.6–0.7. The bacterial culture was kept at 4°C for 30 minutes while the temperature of the shaker was cooled down to 28°C. 0.5 mM isopropyl β-D-thiogalactoside (IPTG) was then added to the culture, and the culture was incubated at 28°C for 3 hours to induce the protein. After harvesting the cells by centrifugation at 4000 rpm for 20 minutes at 4°C, the bacterial pellet was washed with 1X PBS and stored at −20°C for later purification or resuspended in 30 ml of lysis buffer (20 mM HEPES-KOH/7.6, 300 mM potassium acetate, 20% glycerol, 10 mM magnesium chloride, freshly added 1X Halt Protease inhibitor cocktail, freshly added 5 mM β-mercaptoethanol) per 1 L bacterial culture along with 125 μg/ml lysozyme. The culture was then incubated for 20 minutes on ice. The lysate was sonicated with 6 short burst of 20 sec followed by intervals of 40 sec for cooling and centrifuged at 10000 rpm for 20 minutes at 4°C to remove cell debris. The lysate was centrifuged again at 23000 rpm for 60 minutes at 4°C to remove the any leftover cell debris. The lysate was diluted up to 150 ml of lysis buffer.

To equilibrate glutathione agarose beads (Thermo Pierce), 1 ml of the beads (wet bed volume) were washed with lysis buffer twice and carefully poured into a closed column. The beads were then allowed to settle without flow. Once the beads were gravity packed, 100 ml of lysis buffer was run through by gravity flow. The diluted lysate prepared above was loaded onto the equilibrated column and ran at a rate of 30 ml/hr at 4°C. The flow-through was reloaded onto the column and the binding step was repeated twice with a 50 ml/hr flow rate at 4°C. The beads were washed with 150 ml of lysis buffer with 30 ml/hr flow rate at 4°C.

For elution of GST-NEMO, 300 μl of freshly prepared elution buffer (10 mM reduced glutathione, 50 mM Tris-HCl/8.0, freshly added 1X Halt Protease inhibitor cocktail, freshly added 5mM β-mercaptoethanol) was applied using gravity flow and collected in a 1.5 ml microcentrifuge tube. For cleavage of GST by GST- human rhinovirus 3C protease, the beads containing GST-NEMO were resuspended in 9 ml of lysis buffer, transferred to a new 14 ml conical tube, and washed with 10 ml of lysis buffer twice. 100 μl aliquot of the beads (wet bed volume) was transferred to an Eppendorf tube and twice washed with dialysis buffer (50 mM HEPES-KOH/7.6, 100 mM potassium acetate, 20% glycerol, freshly added 5 mM β-mercaptoethanol). 400 μl of dialysis buffer containing 4 μg of GST- human rhinovirus 3C protease was added to the wet beads and the beads were rotated at 4°C overnight. The beads were then centrifuged at 1500 rpm for 20 sec at 4°C to remove GST- human rhinovirus 3C protease and to recover the GST cleaved NEMO protein in the supernatant. To determine the concentration of the purified recombinant NEMO, proteins were analyzed on SDS-PAGE gel together with a serial dilution of BSA at known concentrations. The concentration of GST cleaved NEMO protein was confirmed by absorbance at 280 nm using Nanodrop (Thermo Fisher Scientific Inc.) using the extinction coefficient of 14440 M^−1^ cm^−1^ which was calculated from the NEMO sequence.

### Preparation of GST- human rhinovirus 3C protease

GST- human rhinovirus 3C protease production was induced in BL21 strain *E*. *coli* with 0.5 mM IPTG for 4 hours at 25°C. Cells were collected by centrifugation, resuspended in lysis buffer (1X PBS, 250 mM NaCl, 0.1% Tween-20, 10 mM β-mercaptoethanol, 1 mM PMSF, 1 mM benzamidine), and lysed by sonication with 1 mg/ml lysozyme. Lysate was clarified by centrifugation for 1 hour at 45,000xg and incubated with glutathione agarose beads at 4°C for 1 hour. The beads were washed extensively with lysis buffer. Proteins were eluted with reduced glutathione added to the wash buffer, dialyzed into cleavage buffer (50 mM Tris-Cl pH 8.0, 75 mM KCl, 50% glycerol), and maintained at −20°C.

### Validation of purified recombinant NEMO by MS/MS analysis

A 48 kD protein band from Coomassie blue stained sodium dodecyl sulfate (SDS)-PAGE gel was sliced out, cut into smaller pieces into an Eppendorf tube, and destained by washing three times for 10 min in 200 μl of 50% (v/v) acetonitrile solution containing 50 mM NH_4_HCO_3_. The gel pieces were then reduced by 50 μl of 20 mM dithiothreitol, 100 mM NH_4_HCO_3_ for 30 minutes at 55°C, alkylated in 50 μl of 20 mM iodoacetamide solution, washed with 100% acetonitrile, and dried at room temperature. 10 μl of modified trypsin (Promega, sequencing grade) (100 μg/ml) in 100 mM NH_4_HCO_3_ was added on the dried gel pieces in the Eppendorf tube and incubated at 37°C overnight. The tryptic peptides were injected onto the C18 column (Waters, 100um x 10cm) and eluted with a linear gradient for 60 minutes from 1 to 45% acetonitrile (in water with 0.1% FA) at a flow rate of 500 nl/min. The effluent was electro-sprayed into the LTQ (Thermo Scientific) mass spectrometer. Database search was carried out using both Sequest and X! Tandem algorithms, and identification confidence was calculated by a published method [20]. Human protein sequence database (Uniprot) was used.

### *In vitro* translation and GST-pull down and co-immunoprecipitation assay

Flag-IKKβ/pcDNA3.1(+) was *in vitro* translated with TNT Coupled Reticulocyte Lysate Systems (Promega) as described by the manufacturer using 1 μg of the plasmid and 50 μl final reaction volume. 1/50 of the reaction was separated by 10% SDS-PAGE and verified by immunoblotting with Flag antibody. For GST pull-down assay, 1 μg of GST or GST-NEMO protein was incubated with 1/5 of the translation reaction containing mock or IKKβ in 800 μl of IP buffer (50 mM Tris-HCl, pH7.5, 150 mM NaCl, 1 mM EDTA, 0.25% Triton X-100, 1 mM DTT, 1X Halt Protease inhibitor cocktail). The mixture was rotated for 5 hours at 4°C. 20 μl of glutathione beads was added to the mixture, and the binding mixture was rotated for another 2 hours at 4°C. After washing the glutathione beads 4 times with wash buffer (50 mM Tris-HCl, pH7.5, 300 mM NaCl, 1 mM EDTA, 0.5% Triton X-100, 1 mM DTT, 1X Halt Protease inhibitor cocktail), the GST-NEMO bound IKKβ was analyzed by 10% SDS-PAGE and immunoblotted with Flag antibody. For co-immunoprecipitation analysis, 1 μg of GST or GST-cleaved NEMO protein was incubated with 1/5 of the translation reaction containing mock or IKKβ in 800 μl of the IP buffer containing 2 μg of anti-NEMO antibody. The mixture was then rotated for 5 hours at 4°C and then 20 μl of protein G-sepharose was added to the mixture. The binding mixture was rotated for another 2 hours at 4°C. After 4 times of washing the beads with the wash buffer, the immunoprecipitated proteins were analyzed by immunoblotting as above.

### Cell culture

1.3E2 murine pre-B cells (obtained from Israel’s group [21]) stably expressing 6x Myc-tagged human NEMO (C5) and 70Z/3 cells (TIB-158, ATCC) were cultured in RPMI1640 medium (GE healthcare HyClone) supplemented with 10% (v/v) fetal bovine serum (GE healthcare HyClone), 50 μM β-mercaptoethanol, and antibiotics (100 IU/ml penicillin and 100 μg/mlstreptomycin) as described previously [22]. Human Jurkat T cells (TIB-152, ATCC) were cultured in RPMI1640 medium supplemented with 10% fetal bovine serum and antibiotics as above. Human retinal pigment epithelial (RPE) cells (obtained from Burkard’s group [23]) were cultured in DMEM/F12 medium (GE healthcare HyClone) supplemented with 10% fetal bovine serum and antibiotics as above. Human embryonic kidney (HEK293) cells (CRL-1573, ATCC) and HeLa cells (CCL-2, ATCC) were cultured in DMEM medium (Corning Cellgro) supplemented with 10% fetal bovine serum and antibiotics as described above. All cells were grown at 37°C in 5% CO_2_ humidified incubator (Thermo Scientific) except HEK293 cells, which were grown at 37°C in a 10% CO_2_ humidified incubator on 0.1% (w/v) gelatin-coated dishes. All cells were passaged at least twice weekly before reaching a cell density of 1 x 10^6^ per ml for suspension cells or reaching 90% confluence for adherent cells. Immunofluorescence analysis was done as in [12,13] except for anti-NEMO antibody (FL-419) used for detection of NEMO.

### Generation of NEMO reconstituted 1.3E2 cell clones

1.3E2 cells were reconstituted with wild-type human NEMO as described previously [22]. Briefly, 5 x 10^6^ 1.3E2 cells were electroporated with 40 μg of human NEMO/pcDNA3.1(+), immediately split into three 10 cm dishes to ensure isolation of independent clones, and incubated for 24 hours in growth media. G418 (Mediatech) at 1 mg/ml concentration was then added to select for resistant pools over 4–5 days. Dead cells were removed by using lymphocyte separation medium (Mediatech). The G418-selected clones were isolated (∼40 clones from each of the three pools) and individually placed in each well of a 96-well plate with 0.2 mg/ml G418 for ∼2 weeks before expanding each clone (∼12–20 clones for each pool) for the analysis of NEMO expression by Western blotting with NEMO antibody, using the C5 clone as the standard in each gel. Generally, 2–4 clones from each pool had equivalent NEMO expression as C5. Protein extraction was done by 2x SDS Laemmli sample buffer or using Totex buffer (20 mM HEPES, pH7.9, 350 mM NaCl, 1 mM MgCl_2_, 0.5 mM EDTA, 0.1 mM EGTA, 20% glycerol, 1% NP-40, 0.5 mM DTT, 1X Halt Protease inhibitor cocktail).

### Quantification of cellular NEMO numbers

10^7^ C5 cells were pelleted at 2000 rpm for 2 minutes and resuspended with 10 ml of 1X PBS resulting in a concentration of 10^6^ C5 cells/ml. The resuspended cells were aliquoted to make a series of diluted cells: 1 x 10^4^, 2.5 x 10^4^, 5 x 10^4^, 1 x 10^5^, 2.5 x 10^5^, 5 x 10^5^ and 1 x 10^6^ C5 cells. Purified recombinant NEMO protein (GST cleaved) was also diluted with 1X PBS to make 0.1, 0.5, 1, 5, 10, 50, 100 ng diluted protein series. 2x SDS Laemmli sample buffer was added to the diluted cells or recombinant NEMO protein. The samples were boiled for 5 minutes and separated on 10% SDS-PAGE gels. The separated proteins were transferred onto polyvinylidene difluoride (PVDF) membranes and visualized by Western blotting with anti-NEMO antibody.

To calculate the number of NEMO molecules per cell on average, the intensity of visualized NEMO bands were measured by densitometry analysis. The intensity of bands at the linear range, corresponding for 1, 5, and 10 ng of the purified recombinant NEMO protein and 5 x 10^4^, 1 x 10^5^, 2.5 x 10^5^ C5 cells, were normalized to the intensity of band corresponding to 1 ng of the purified recombinant NEMO protein. A graph and equation were achieved from the normalized intensity to estimate the number of NEMO molecules per cell on average. Based on this equation, the intensity for 1 x 10^5^ C5 cells was equivalent to the intensity of band corresponding to 4.55 ng of the purified recombinant NEMO protein. Using molecular weight of 6x myc tag-human NEMO (62.6 kDa), 4.55ng equals to 72.7 fmoles or approximately 4 X10^10^ NEMO molecules, indicating approximately 4 x 10^5^ molecules of human NEMO protein is expressed in a single C5 cell on average. Three independent analyses were performed by three investigators on different days with the same outcomes obtained (with R^2^ value ranging from 0.92 to 0.99).

To quantify the number of NEMO molecules in different human cell lines, 3 x 10^6^ cells each of Jurkat, HEK293, HeLa, or RPE, and C5 were pelleted at 13,000 x g for 10 s in a table-top microcentrifuge at room temperature, rinsed once with phosphate-buffered saline (PBS), resuspended in 50 μL PBS, and lysed by the addition of 250 μL 2x SDS Laemmli sample buffer. Samples were then immediately boiled for 10 minutes, loaded by cell number in a titration from 10^6^ to 10^4^ cells, and separated on 10% SDS-PAGE gels. The separated proteins were visualized by Western blotting with anti-NEMO antibody as described above. Densitometry analysis using ImageJ software was used to measure the intensity of the visualized NEMO bands for each blot, and the signals were then normalized to either 2.5 x 10^5^ or 5 x 10^5^ of corresponding C5 cells for each blot. Using the normalized intensities, a linear graph and equation were generated for each cell type with R^2^ value > 0.94. The average number of NEMO molecules per cell was then determined by calculating the ratio of the slopes between each cell type Jurkat, HEK293, HeLa, or RPE, and C5 and multiplying this ratio by the estimated average number of NEMO molecules per C5 cell.

### Statistical analysis

Values are presented as means ± S.E.M. with the indicated number of independent experiments. The statistical significance of differences between groups was determined by the Student's t test. P values less than 0.05 were considered statistically significant.

## Results

### Purification of full-length NEMO

**Fig 1:**
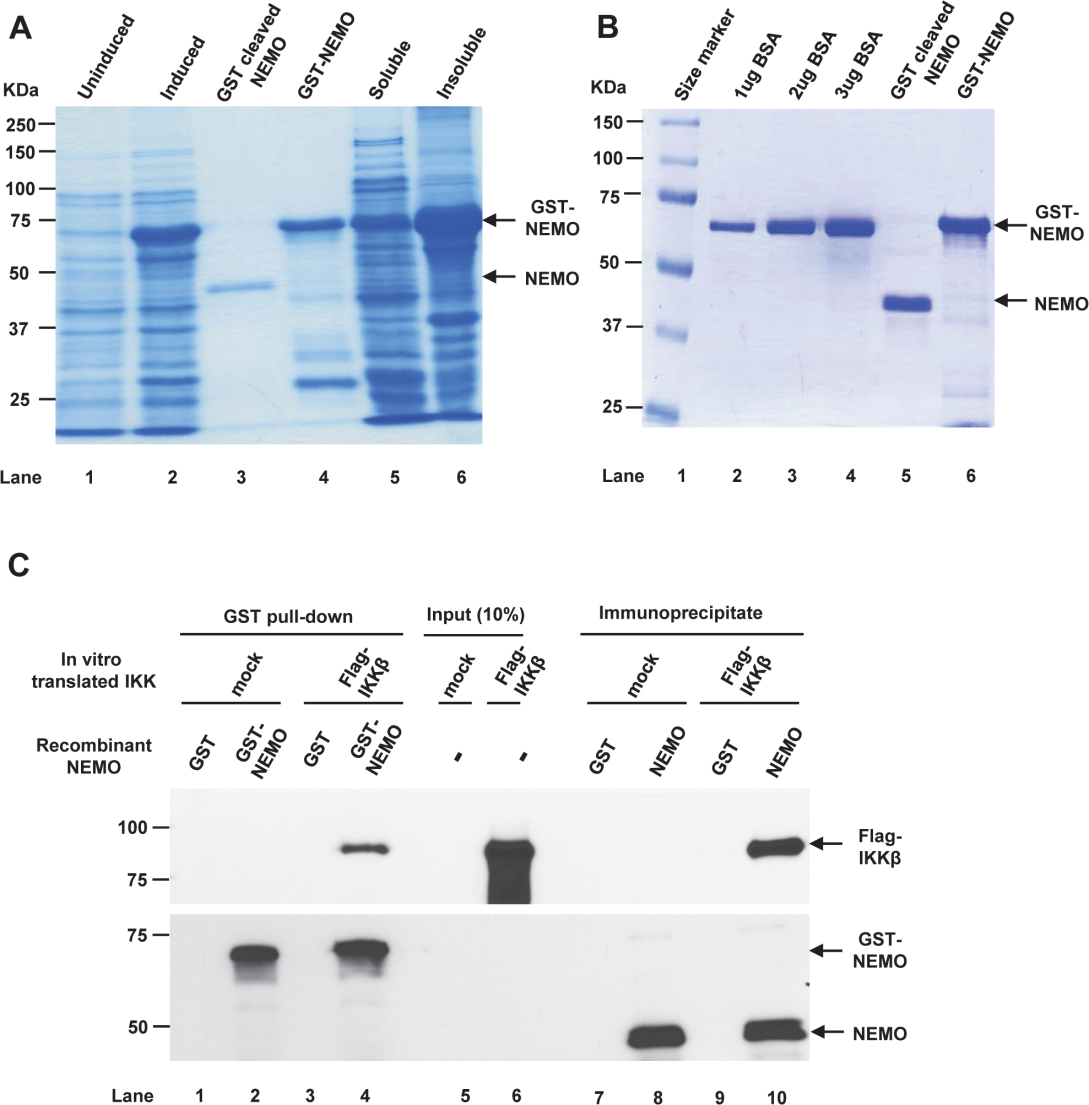
Purification of full length recombinant human NEMO protein. (A) SDS–PAGE gel stained with Coomassie Brilliant Blue R-250 for the protein purification profile. (B) 10 μl of purified recombinant NEMO proteins were loaded with a serial dilution of BSA at known concentrations (C) GST or GST-NEMO protein was incubated with *in vitro* translated mock or IKKβ for GST pull-down assay (lane 1–4), or GST or GST-cleaved NEMO protein was incubated with *in vitro* translated mock or flag-IKKβ for co-immunoprecipitation with anti-NEMO antibody (lane 7–10). Flag-IKKβ in the complexes or inputs (lanes 5 and 6) were separated on SDS-PAGE gel and immunoblotted with flag antibodies.

To purify sufficient amounts of full-length recombinant human NEMO protein, a human NEMO construct was transformed into Rosetta2 BL21 cells and the NEMO protein was induced. Regardless of different induction temperatures (19–30°C), at least 60% of the induced NEMO protein was found in the insoluble fraction (Fig. 1A, lane 6). To reduce the aggregation of the NEMO protein and maximize the yield of NEMO protein in the soluble fraction, the bacterial lysate was diluted with a large volume of lysis buffer (150 ml per 1 L of the bacterial culture). The diluted lysate was slowly loaded onto a glutathione column with three repeated applications to further reduce the potential of NEMO aggregation. In addition, β-mercaptoethanol freshly added to all buffers increased the yield of the soluble NEMO protein, possibly by further preventing protein aggregation [19].

Despite extensive washing with lysis buffer, the GST-NEMO fraction, which was eluted with elution buffer containing 10 mM reduced glutathione, contained nonspecifically bound bacterial proteins (Fig. 1A, lane 4). However, direct cleavage of GST-NEMO from GSH-beads with GST-tagged human rhinovirus 3C protease resulted in the recovery of cleaved NEMO protein fractions that contained significantly reduced protein contaminants compared to the GST tagged NEMO fraction (Fig. 1B, lane 5 and 6). Even so, the NEMO protein was extremely susceptible to aggregation after the GST tag was removed. Thus, it was important to maintain at least two bead volumes of cleavage buffer to prevent NEMO protein aggregation. Approximately 0.3 mg/ml of GST-cleaved full length NEMO and 1 mg/ml of GST-NEMO in a soluble state could be obtained.

The nature of the purified NEMO protein was confirmed by excising ∼48 kD protein band from Coomassie blue-stained SDS-PAGE gel followed by MS/MS analysis. Sequence coverage with high confident peptides was 55.8% (S1 Fig.). Moreover, both purified GST-NEMO and GST-cleaved NEMO bound to *in vitro* translated flag-IKKβ by GST-pull down assay and co-immunoprecipitation assay, respectively (Fig. 1C). These results confirmed the purity and identity of the purified recombinant NEMO protein, which was used for subsequent cellular NEMO quantification studies.

### Determination of the average number of NEMO molecules in the C5 clone

**Fig 2:**
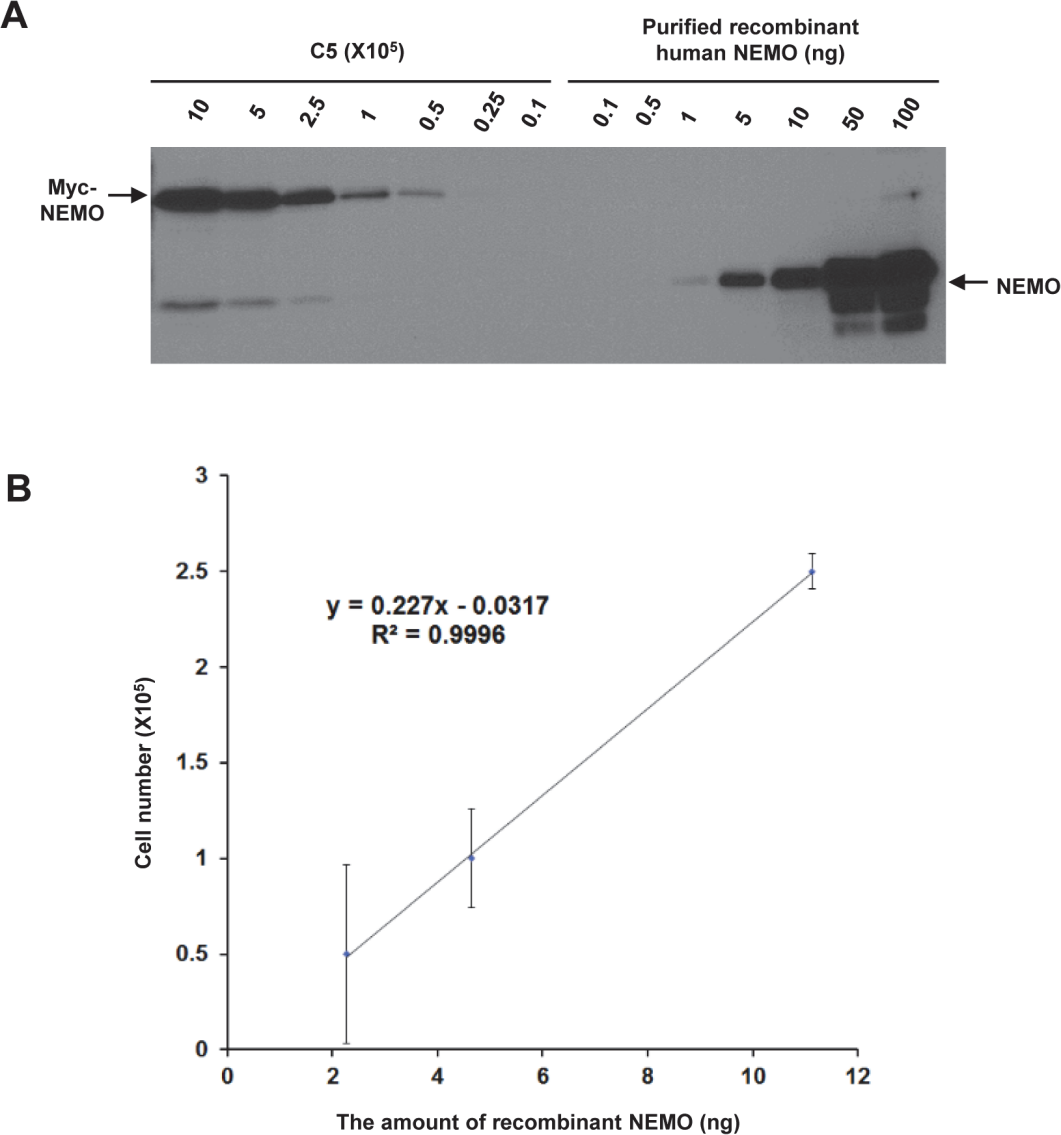
Determination of the average number of NEMO molecules in the C5 clone. (A) Protein extracts from the indicated number of C5 cells and indicated amount of purified recombinant human NEMO proteins were separated on SDS-PAGE gel and immunoblotted with NEMO antibody. (B) Graph representing the intensity of the bands corresponding to NEMO in three independent replicates as shown in (A) and an equation derived from the graph. Error bars represent S.E.M.

To estimate the average number of NEMO molecules present within a cell, we employed a 1.3E2 clone (C5) which stably expresses 6x Myc-tagged NEMO at approximately the same levels as endogenous NEMO levels detected in 70Z3 cells, the parental cell line of 1.3E2, using FL419 NEMO antibody. C5 and 70Z/3 cells display similar levels of NF-κB activation in response to etoposide (VP16) and lipopolysaccharides (LPS; membrane signaling control) [13]. Different amounts of purified recombinant NEMO protein along with cell extracts representing different C5 cell numbers were analyzed by Western blotting using anti-NEMO antibody (Fig. 2A and S2 Fig.). Based on quantification of the intensity of the NEMO bands through densitometric analysis, approximately 4 x 10^5^ molecules of human NEMO protein is expressed in a single C5 cell on average (Fig. 2B).

### Generation of 1.3E2 clones expressing different amounts of NEMO protein

**Fig 3:**
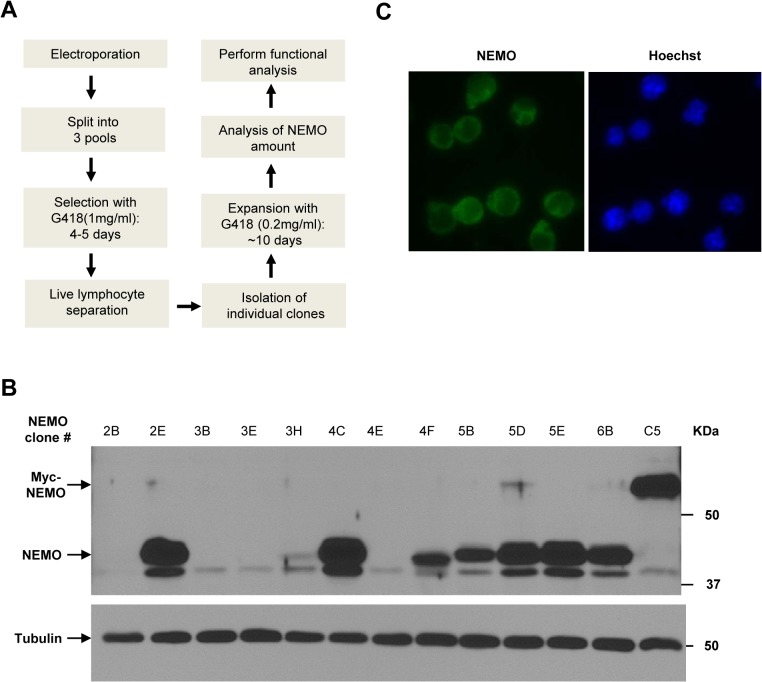
Generation of 1.3E2 clones expressing different amounts of NEMO protein. (A) Work flowchart to generate NEMO reconstituted 1.3E2 cell clones. (B) An example of Western blots with different NEMO stable clones. Protein extracts from different 1.3E2 clones were separated on SDS-PAGE gel and immunoblotted with NEMO antibody. Tubulin was used as a loading control. (C) An example of immunofluorescence analysis with NEMO antibody (Green) in a NEMO reconstituted 1.3E2 stable clone. Nuclei were visualized by Hoechst staining (Blue).

To determine the impact of different NEMO expression levels on NF-κB activation by genotoxic agents, we stably reconstituted NEMO in 1.3E2 cells with tag-less human NEMO as depicted in Fig. 3A. An example of the Western blot analysis of 1.3E2 cell clones stably expressing different amounts of NEMO is shown in Fig. 3B. Based on the expression of NEMO relative to the C5 clone, multiple independent stable clones were further expanded in order to determine the average number of NEMO expression per cell and their respective NF-κB activation potentials in response to etoposide treatment. When different clones were analyzed by immunofluorescence analysis using anti-NEMO antibody, the expression of NEMO detected in individual cells was relatively uniform (Fig. 3C).

**Fig 4:**
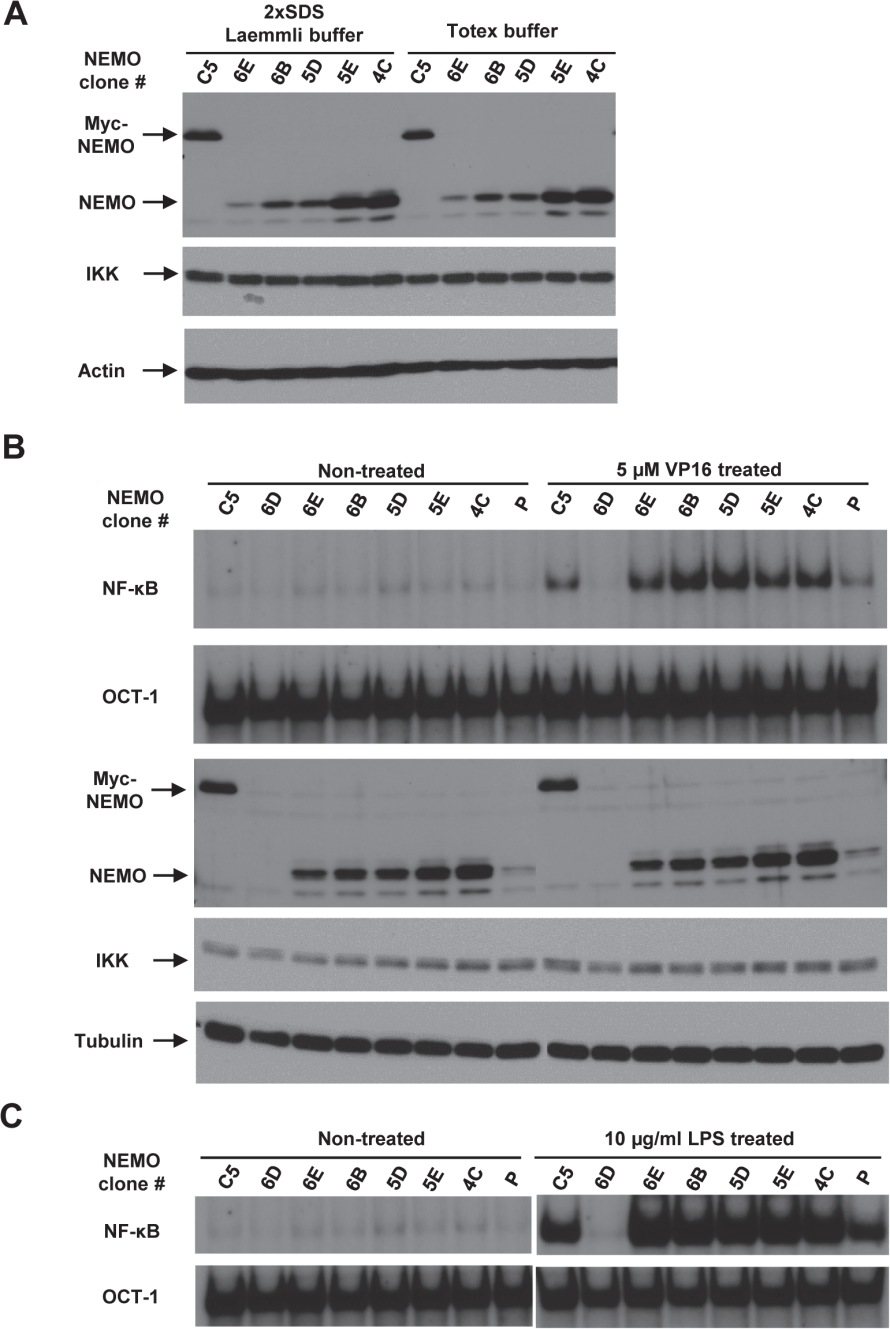
Effects of NEMO amount on NF-κB activation. (A) Cell extracts from equal numbers of 1.3E2 clones expressing different amounts of NEMO protein were generated using either 2x SDS Laemmli sample buffer or Totex buffer. The cell extracts were separated on SDS-PAGE gel and immunoblotted with NEMO, IKKα/β, and actin antibodies. (B, C) The 1.3E2 clones as in A were left untreated or treated with etoposide (VP16, 5 μM, 1 hr) in B or LPS (10 μg/ml, 30 min) in C. P represents pool of the 1.3E2 clones before isolation of individual clones. Protein extracts were examined by EMSA and Western blotting using NEMO, IKKα/β, and tubulin antibodies.

We next treated multiple 1.3E2 clones, stably expressing different levels of NEMO, with etoposide for 1 hour and analyzed NF-κB activation by EMSA. Since NEMO is prone to precipitate *in vitro* [19], the amount of NEMO in the clones was measured using two different extraction methods: 2x SDS Laemmli sample buffer and Totex buffer, the latter of which was used to prepare cell extracts for EMSA assay as NF-κB proteins are efficiently extracted in the native state. Amounts of extracted NEMO were similar in both methods for tag-less NEMO (Fig. 4A).

**Fig 5:**
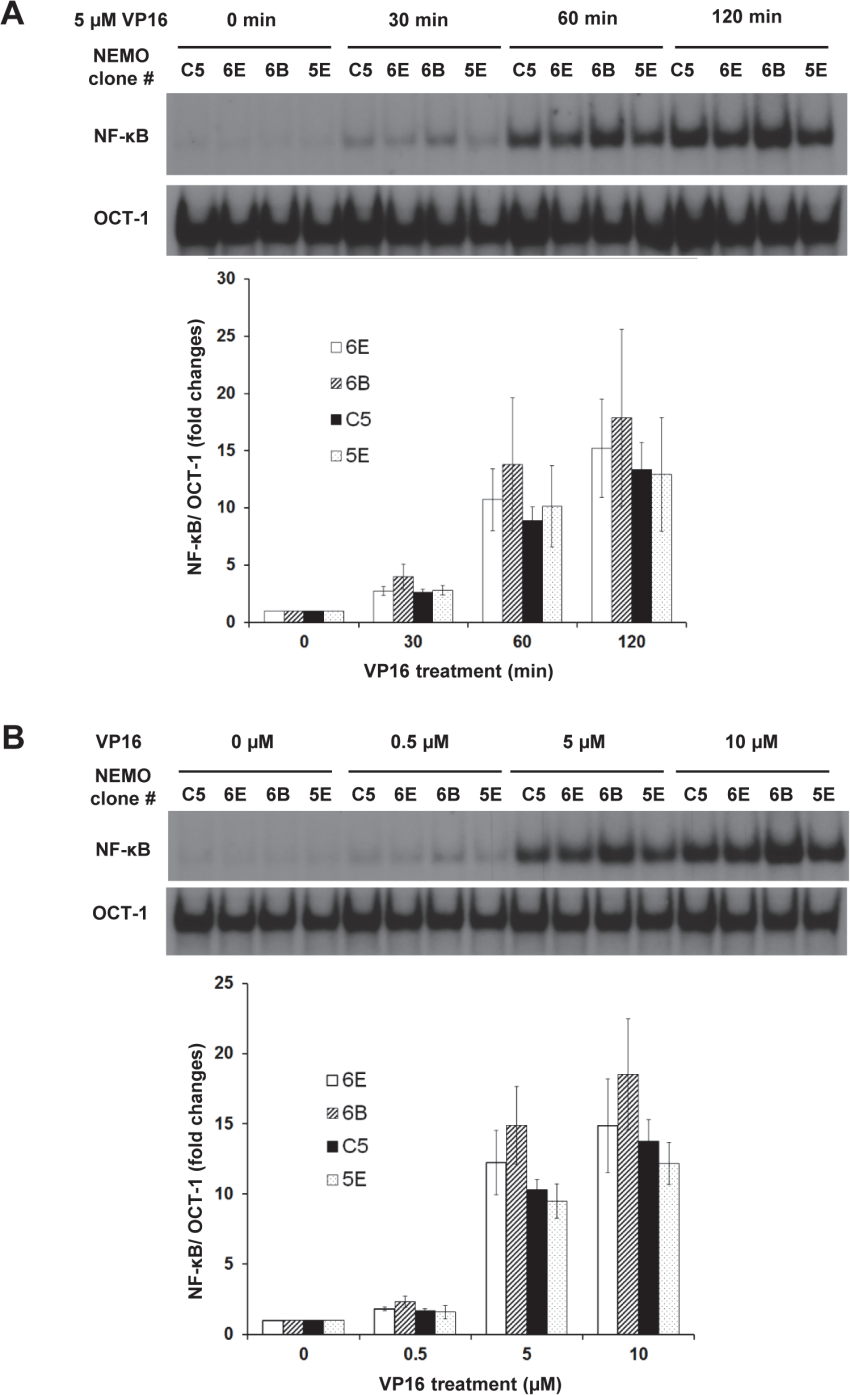
Effects of NEMO amount on NF-κB activation in response to etoposide. (A) Time course analysis. The indicated 1.3E2 clones were left untreated or treated with 5 μM VP16 for indicated times and analyzed by EMSA. Three independent experiments were performed as above on different days. The NF-kB bands were quantified and fold inductions of NF-κB DNA binding activity were graphed below. Error bars represent S.E.M. (B) Dose response analysis. The indicated 1.3E2 clones were left untreated or treated with the indicated concentration of etoposide (VP16) for 1 hr. The quantification and graph were done as in A.

To estimate the number of NEMO molecules in the NEMO reconstituted clones shown in Fig. 4A, the equation derived in Fig. 2B and a comparison of the NEMO bands between the clones and C5 were used. Based on our calculations, we estimated that clones 6E, 6B, 5D, 5E and 4C express 0.6, 2.7, 2.7, 6 and 7 x 10^5^ molecules of NEMO per cell. All of these NEMO clones showed almost no detectable basal NF-κB activity (Fig. 4B). In response to etoposide treatment, a trend of increasing NF-κB activation was observed as the amount of NEMO increased (peaking with clone 6B), which became modestly reduced in clones which expressed even higher amounts of NEMO (Fig. 4B). In contrast, the NF-κB activation in response to LPS was not affected by the amount of NEMO in this range (Fig. 4C). More careful analysis using both a time course (Fig. 5A) and dose response (Fig. 5B) showed similar trends with clone 6B showing the highest NF-κB activation and lower or higher amounts of NEMO than clone 6B showing reduced NF-κB activation. However, quantification of EMSA data from multiple biological replicates did not show statistically significant impacts (Fig. 5A and 5B, lower graphs). Thus, NF-κB activation by etoposide is surprisingly invariant across a 10-fold range of NEMO expression in the 1.3E2 cell system.

### Determination of the average number of NEMO molecules per cell in multiple human cell lines

**Fig 6:**
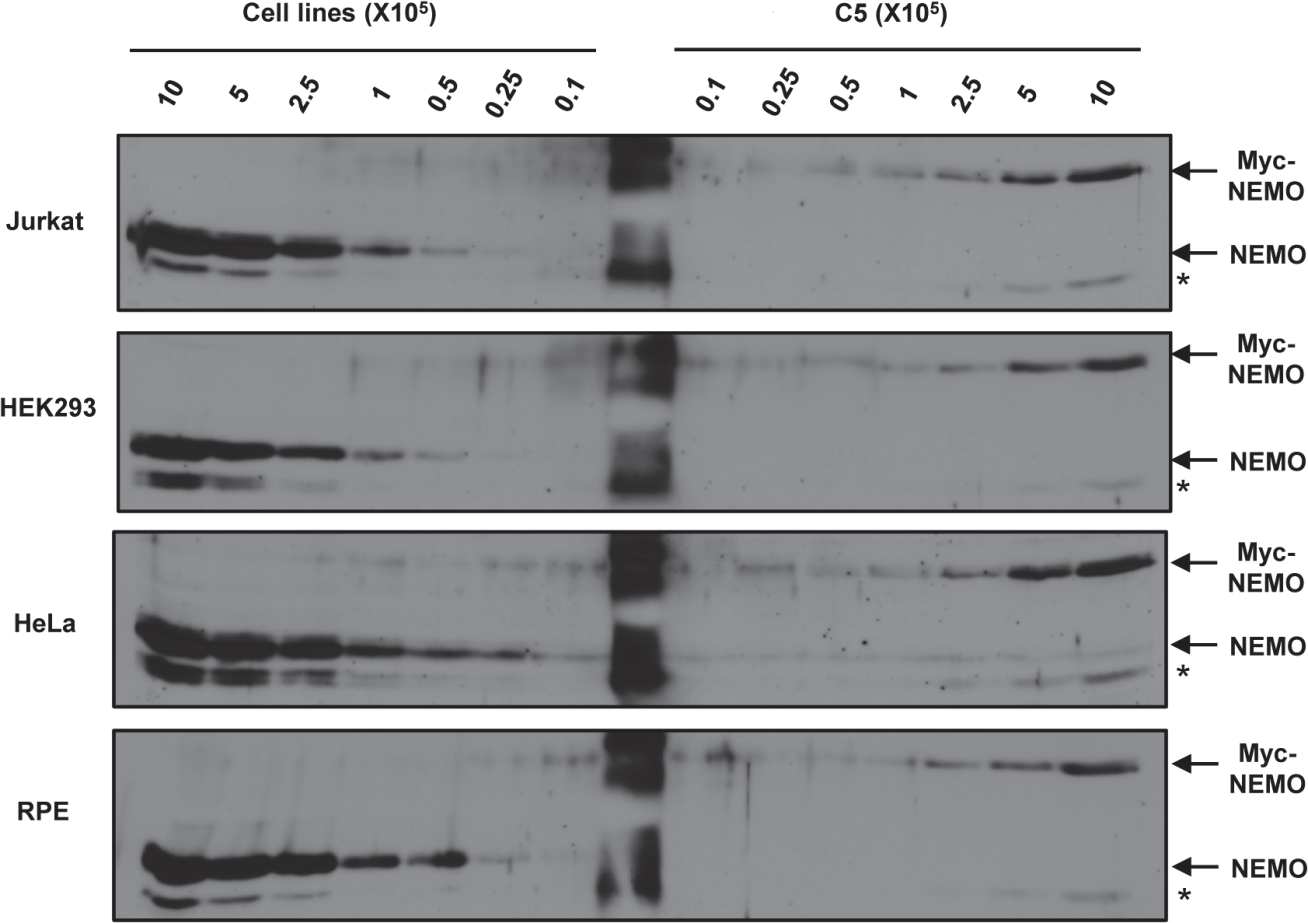
Determination of the average number of NEMO molecules in multiple human cell lines. Representative NEMO Western blots of protein extracts from the indicated number of Jurkat, HEK293, HeLa, and RPE cells compared to protein extracts from the indicated number of C5 cells are shown. * represents non-specific band. Each blot was performed three biological replicates except for HEK293 which was two biological replicates.

Various human cell lines are often employed to study NF-κB signaling by different stimuli, including genotoxic agents; however, the number of NEMO molecules expressed per cell in any human cell line remains undefined. Using the C5 clone as a standard, we sought to estimate the average number of NEMO molecules in the following human cell lines: Jurkat T lymphocytic, HEK293 embryonic kidney, HeLa cervical, and RPE retinal pigment epithelial cells. Cell extracts of each cell line, as well as those of the C5 clone, were prepared using 2x SDS Laemmli buffer and analyzed by Western blotting using anti-NEMO antibody (Fig. 6). Quantification of the NEMO band intensities yielded the following estimates for the average number of NEMO molecules per cell for each cell line: Jurkat—1.3 x 10^6^; HEK293–1.0 x 10^6^; HeLa—1.0 x 10^6^; RPE—2.0 x 10^6^ (Table 1). All of these human cell lines are considerably larger than 1.3E2 pre-B cells, which may account for the relatively larger average numbers of NEMO molecules per cell.

**Table 1:** Average number of NEMO molecules per cell of various cell lines compared to C5.

Cell Line	Ratio to c5	Average # NEMO molecules/cell
**Jurkat**	3.1 ± 0.2	1.3 x 10^6^
**HEK293**	2.5 ± 0.1	1.0 x 10^6^
**HeLa**	2.5 ± 0.2	1.0 x 10^6^
**RPE**	5.6 ± 0.1	2.0 x 10^6^

Biological replicates were performed as such: Jurkat, HeLa, and RPE: n = 3, HEK293: n = 2.

## Discussion

In previous studies, investigation of the functional significance of NEMO and its mutants in multiple NF-κB signaling pathways utilized reconstitution of NEMO mutants in NEMO-deficient 1.3E2 murine pre-B cells, R5 rat fibroblast, and Jurkat human T cells, or NEMO-null mouse embryo fibroblasts [12,21,24,25]. The interpretation of the functional significance of mutant NEMO proteins is confounded by the potential artifacts associated with their overexpression as overexpression of even the wild-type NEMO protein can significantly inhibit NF-κB signaling [16–18]. Indeed, while NEMO was originally cloned through functional restoration of NF-κB signaling by its stable reconstitution in NEMO-deficient R5 and 1.3E2 cells [21], it was also independently cloned by another group as IKK/NF-κB inhibitor protein upon its overexpression [26]. One approach to control the expression of NEMO mutants is to generate inducible cell systems (e.g., doxycycline-inducible system) or use of knock-in technologies (e.g., CRISPR) [27,28]. Alternatively, co-expression of a marker protein (e.g., CD2 or GFP) as a NEMO fusion or expression from an internal ribosome entry sequence within a single expression plasmid may be used as a surrogate marker of expression quantified and isolated by FACS sorting [18]. Even with these approaches, it may be necessary to ensure the expression level of NEMO to be "physiological" by comparing the expression of mutant NEMO proteins to the endogenous levels of the parental cell systems as comparison to wild-type NEMO protein may be insufficient to draw accurate conclusions if the wild-type NEMO expression levels are highly overexpressed compared to the endogenous levels.

We have previously employed stable 1.3E2 cell clones to study NEMO mutant phenotypes in NF-κB signaling induced by genotoxic agents [12,13,29]. The expression of NEMO in stable 1.3E2 clones remains generally stable over many passages and cryopreservation steps, and individual cells within a given clonal population generally express similar levels as assessed by immunofluorescence analysis (e.g., Fig. 3C). In contrast, expression of NEMO in individual cells of a stable pool population is typically highly variable, making it a generally unsuitable system to study NEMO mutant phenotypes as the NF-kB activation in those individual cells with very low or high NEMO expression will likely show different phenotypes as those with more physiological NEMO expression levels within a stable pool. However, it is technically challenging to match the expression levels of NEMO in stable clones across different mutant NEMO versions. Our current study demonstrates that the levels of NEMO do not have to be precisely matched to draw functionally relevant conclusions in the context of 1.3E2 reconstitution system. Based on our quantification analysis, using the C5 stable clone that expresses approximately 4 x 10^5^ molecules of human NEMO protein per cell as a standard, we found that a range of 0.6–6 x 10^5^ NEMO molecules per cell (a 10-fold range) is functionally equivalent with statistically insignificant impact on etoposide-induced NF-κB activation as measured by EMSA analysis.

Since our previous studies suggested that only “IKK-free” NEMO, which is not bound to IKKα/β, mediates nuclear signaling in response to genotoxic agents [12], we originally considered the possibility that the increased amounts of NEMO beyond some threshold level (perhaps set by the levels of IKKα/β subunits) might increase the level of NF-κB activation in response to genotoxic agents by increasing free NEMO. Indeed, there was a trend of modest increase in NF-κB activation by etoposide as the level of NEMO increased from clone 6E to 6B/5D (Fig. 4A and 4B). As the level of NEMO further increased, etoposide-induced NF-κB activation was modestly reduced and a greater decrease in NF-κB activation was found in some clones that expressed an amount of NEMO beyond this range (data not shown). Despite the varied NEMO levels from undetectable to that which is much higher than the C5 clone, the amount of IKKα/β expression was invariant, suggesting that the levels of IKK catalytic subunits are not regulated by NEMO protein in this cell type. Thus, estimation of the number of IKKα/β molecules per 1.3E2 cell and the definition of the stoichiometry of NEMO and IKKα/β molecules would be informative for further elucidation of the mechanisms involved in NF-κB activation by genotoxic agents. It is also possible that "IKK-free” NEMO is liberated from NEMO-IKK complexes upon stimulation with genotoxic agents and therefore the basal stoichiometry of NEMO and IKKα/β molecules may be insufficiently informative. As such, additional studies aiming at defining the dynamics of NEMO-IKKα/β complexes during cell stimulation may also be critical to improve our understanding of NF-κB signaling by DNA damaging agents.

The level of NEMO expression in the C5 clone was stable over multiple cryopreservation cycles and over multiple independent analyses; therefore, it may be useful as a standard cell line to estimate the number of NEMO molecules in other human cell types as we have done so for several commonly used human cell lines. However, we cannot rule out the possibility of some posttranslational modifications of NEMO altering the degree of epitope recognition by anti-NEMO antibody. The cell lines derived from non-human species may also need to be recalibrated by the use of recombinant full-length NEMO protein standards generated from the species of interest as different anti-NEMO antibodies likely recognize NEMO proteins from different species with varying efficiencies depending on the conservation of the epitopes being detected.

As part of the NEMO quantification studies, we generated recombinant full-length human NEMO protein to be used as a molecular standard. Even though multiple groups have previously purified recombinant NEMO protein for biochemical study, the recombinant NEMO proteins were generally purified as fusion proteins [19,30–32] and we were unable to find a study that reports a purification protocol for untagged full-length recombinant wild-type NEMO protein. Therefore, we sought to optimize a NEMO purification protocol to obtain relatively pure and concentrated amounts of the soluble, full-length, and untagged NEMO protein. A well-appreciated obstacle in the purification of recombinant full-length NEMO protein is protein aggregation and precipitation. While the NEMO protein was more soluble as GST-fusion protein, it became more susceptible to aggregation when the GST was removed from the protein, suggesting GST-induced dimerization of NEMO potentially reduces NEMO aggregation at high concentrations. Agou et al. suggested the use of neutral detergents such as dodecyl maltoside to prevent this aggregation, however this increased the co-purification of bacterial DnaK protein with NEMO protein and thus decreased overall purity [30]. Whitty and colleagues have recently generated an N-terminal seven cysteines-to-alanines substitution mutant of NEMO to increase the purification of soluble NEMO protein by preventing intermolecular disulfide bond formation [19]. Another possible way to increase the solubility of NEMO protein is to co-purify NEMO with other proteins that interact with NEMO to prevent NEMO aggregation, such as the IKK catalytic subunits [32]. We found that keeping the bacterial lysate dilute in large volumes and adding a fresh reducing regent to all the buffers increased yield of soluble NEMO protein. Moreover, direct cleavage of GST-NEMO proteins from the GSH-beads by GST-tagged human rhinovirus 3C protease in large reaction volumes also resulted in isolation of soluble NEMO with high purity and at a maximal concentration of 0.3 mg/ml. Further concentration of NEMO resulted in aggregation and precipitation under our current buffer conditions. The identity of purified NEMO protein was validated by MS/MS analysis as well as by IKKβ interaction *in vitro* and association with appropriate ubiquitin chains (data not shown). The NEMO purification protocol described here thus may be useful for purification of non-human NEMO proteins as well as future *in vitro* studies of NEMO in NF-κB signaling.

## Supporting Information

S1 Fig Protein sequence coverage of human NEMO sequence by MS/MS analysis. Matched peptide sequences are shown in yellow in the diagram and red in amino acid sequences.
(TIF)

S2 Fig Determination of the average number of NEMO molecules in the C5 clone. Narrowed amount range of purified recombinant human NEMO proteins were analyzed with protein extracts from the indicated number of C5 cells.
(TIF)
