# Supplementary figures and images for: Quantification of Cellular NEMO Content and Its Impact on NF-κB Activation by Genotoxic Stress
Source: PLoS One. 2015 Mar 5;10(3):e0116374. doi: 10.1371/journal.pone.0116374 (PMC4350935; doi:10.1371/journal.pone.0116374)

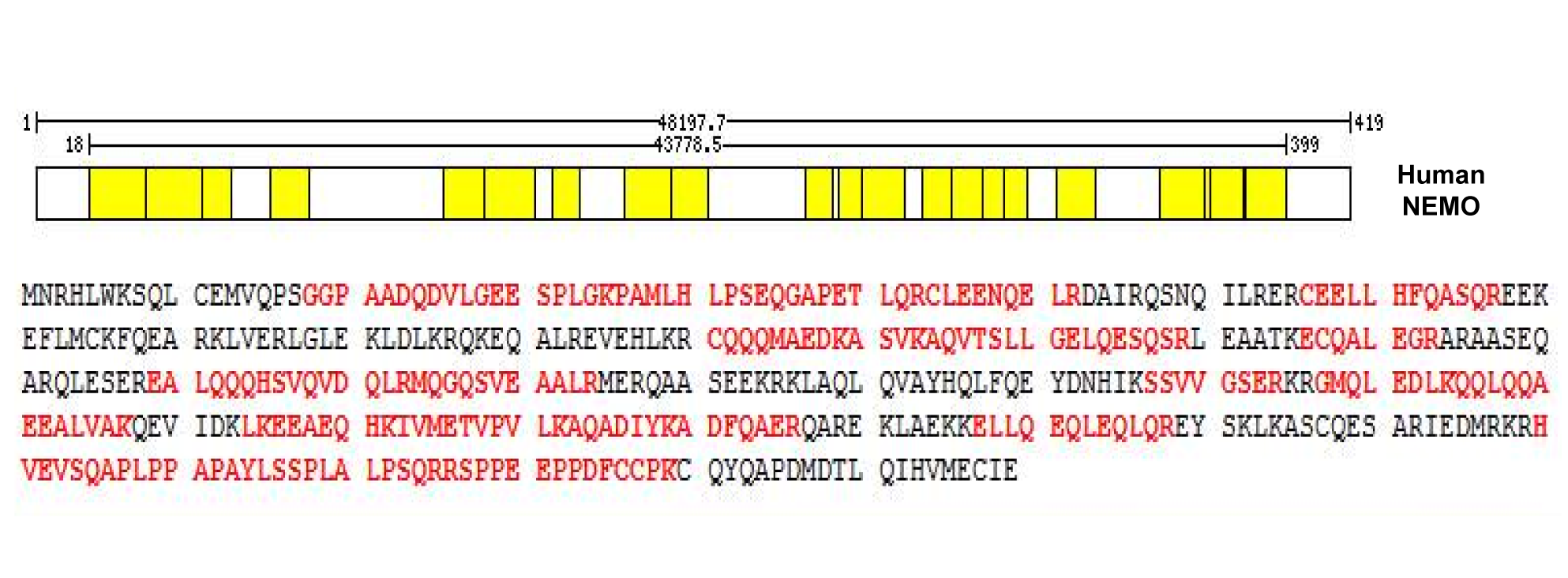

Supplement: S1 Fig — Matched peptide sequences are shown in yellow in the diagram and red in amino acid sequences. (TIF) [file pone.0116374.s001.tif]

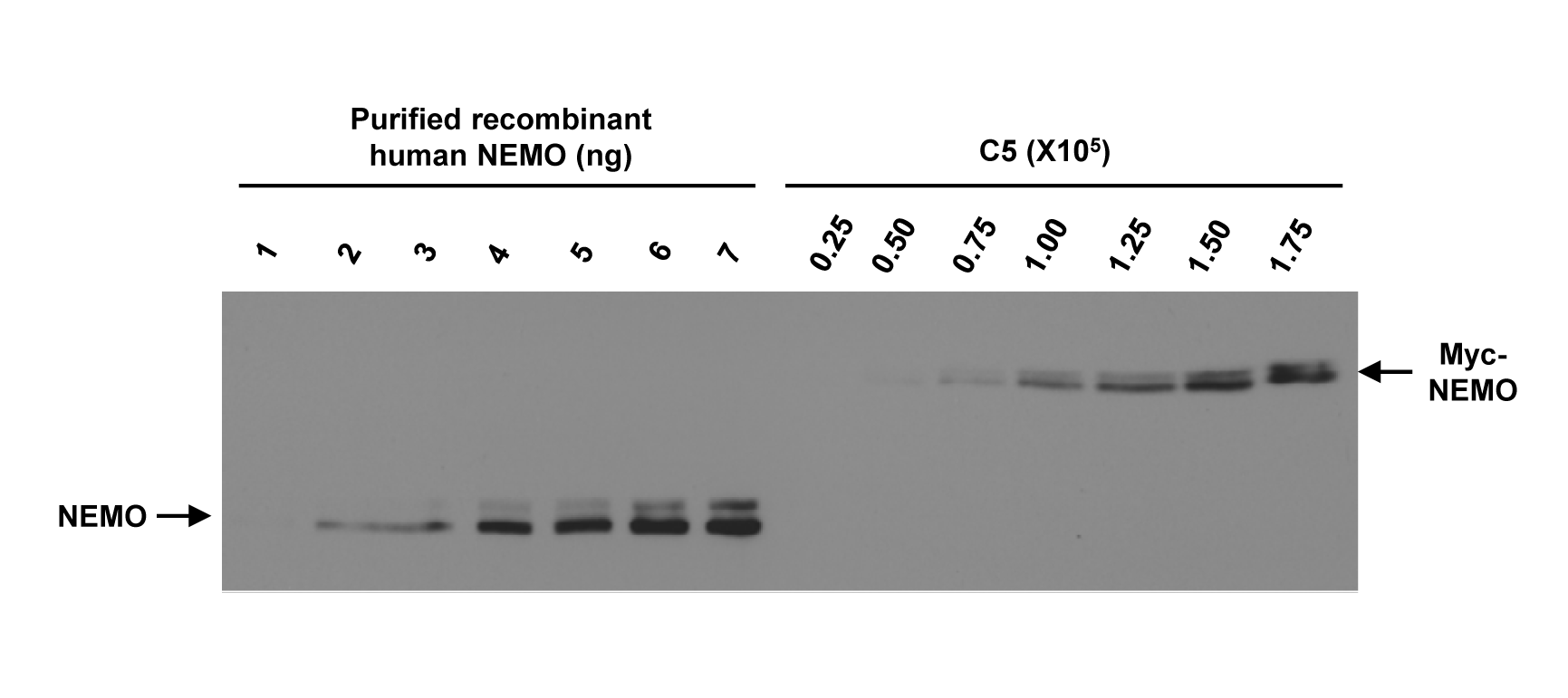

Supplement: S2 Fig — Narrowed amount range of purified recombinant human NEMO proteins were analyzed with protein extracts from the indicated number of C5 cells. (TIF) [file pone.0116374.s002.tif]
